# Navigating challenges and opportunities: perspectives on digital service development in substance use disorder treatment

**DOI:** 10.1186/s13011-024-00618-6

**Published:** 2024-08-01

**Authors:** Janika Kosonen, Gillian W. Shorter, Katja Kuusisto

**Affiliations:** 1 Tampere University, Tampere, Finland; 2 Queen’s University Belfast, Belfast, Northern Ireland UK

**Keywords:** Substance use, Substance use disorder, Addiction, Digitalisation, Health care, Social work, Stigma

## Abstract

**Background:**

Some people with substance use disorders (SUD) can experience multiple co-occurring social problems. Digital solutions have been developed to support effective and cost-effective social welfare and healthcare in addictions treatment. Given the varying severity of problems from alcohol and other drug use, digital service tools can save money and provide tailored care.

**Objective:**

In this study we aimed to understand the perspectives of those who develop digital service tools on people with SUD and treatment encounters. As a case, we interviewed those who have been involved in the development of a digital client segmentation tool *The Navigator.*

**Methods:**

Ten (*N* = 10) semi-structured interviews were conducted with professionals involved in digital client segmentation tool development and were analysed with inductive content analysis. Participants were asked about the development of the Navigator from the perspectives of their own role as developers, the clients, the effectiveness of the services, and decision-making processes.

**Findings:**

Some people with SUD may face several obstacles when using digital services. Digital divide, feared or experienced stigma and biased attitudes, complex life situations, and difficulties in committing to treatment were identified as challenges. Nevertheless, digital solutions can offer the clients alternative ways of using the services that can better meet their individual needs. The anonymity and facelessness of digital solutions can reduce the fear of immediate judgement. Implementing digital solutions in substance use work poses challenges due to chronic staff shortages. Digitalisation often results in the creation of multiple simultaneously managed channels, potentially reducing time-consumption but increasing the perceived workload. There is a call for multi-professionalism, acknowledging inequalities between various disciplines within the field.

## Background

Digitalisation is becoming increasingly common in social welfare and healthcare services, shaping the methods of receiving and accessing care, treatment, and services [1]. The increasing use of digital transactions in social welfare and healthcare systems requires careful consideration of the individual needs of service users [2]. Internationally, the adoption of digital solutions has been accelerated by the COVID-19 pandemic [3, 4]; the pandemic has also influenced the recovery processes of those who recover from substance use disorders (SUD) [5]. As innovation is integrated into routine practice, it is essential for statutory services with regulatory functions to ensure they meet the needs of service users and provide the entitled support.

Some people with substance use problems can experience co-occurring psychosocial issues such as homelessness or vulnerable housing, problems within social networks, experiences within the criminal justice system, experiences of different forms of violence, abuse and/or other trauma histories, and/or mental health issues (see e.g., [6–9]). Substance use can be associated with shame and stigma from self and others and increase the barriers to seeking help if it is needed [8, 10]. This judgement can also originate from professionals who have the job of caring for those with SUD [11, 12].

Digital solutions for addiction care and treatment can have both positive and negative effects on clients and those who care for them. Positive effects can include e.g., reduced stigmatisation, increased addiction identification, better cost-efficacy and cost-control and lower-threshold access to services [13–15]. However, digital interventions seem to best serve those whose life situations, experienced challenges and use of intoxicants are under control and not yet escalated since they tend to require a certain level of self-direction and independency [14]. Also, reduced costs are not self-evident, and sometimes the adherence maintenance costs can even equal face-to-face treatment [16].

Digital innovations are not able to resolve the issue of poor availability or resources of addiction services, and they should not replace but rather supplement traditional face-to-face encounters [14, 16]. *Digital divide* means unequal access to digital devices and services which dichotomously divides people into those who have the access and to those who do not [17]. In the era of rapid digitalisation, despite the ethical, legal, regulatory and social challenges, there is a great need for digital interventions for stigmatised groups, including people with SUD [15]. The development of digital innovations for these target groups requires user-involvement, and robust evaluation and evidence base [15, 18]. However, the current evidence base of digital interventions for assessment and case management in addiction treatment appears to be weak and under-developed [15]. Nonetheless, online therapy interventions have shown promising results in addiction treatment for those who can access them [19–22].

In this study, we are interested in exploring how professionals involved in the development of digital solutions for social welfare and health care purposes perceive the compatibility of digital service tools for the care of people with SUD. As a case, we interviewed professionals involved in the development of a Finnish digital client segmentation tool called *the Navigator*. The Navigator was originally developed for healthcare purposes in Finland while there have been efforts to expanding it to social work purposes. Within the Navigator, client segmentation occurs during client-professional interaction, in the service needs assessment phase, and the outcome is based on both the client’s and the professional’s perceptions of the client’s service needs. The Navigator has four different client groups or segments, aiding delivery of appropriate services to the client. The Navigator has proven feasible for diabetes patients during nurses’ appointments in primary care [23].

Digital client segmentation is a well-established practice in other industries e.g. [24, 25] often developed to assist businesses in finding profitability and potential [26]. For social welfare and healthcare purposes, digital client segmentation, or stepped care, has been adapted to enhance cost-efficiency and improve the quality of treatment [22]. The premise and objectives of digital client segmentation in social welfare and healthcare reveals its prevailing neoliberal administrative ideas in practice. According to Engström and colleagues [25], these objectives encourage service providers to standardise rather than personalise service delivery, consequently increasing the risk for the most vulnerable clients to be excluded from the service system. Lynn and colleagues [24] criticized stepped care for its service provider orientation and promoted for client-oriented segmentation instead.

Digitalisation of social welfare and healthcare raises concerns about access and equity, from individual-level obstacles to systemic issues [23, 24]. In this study, we aimed to explore the opportunities and challenges of digital service development for people with SUD based on the case *the Navigator*. We explored *how professionals involved in the development of the Navigator perceive 1) individuals with SUD and 2) substance use workers as the end users of digital solutions?* In reporting this study, we used the Consolidated Criteria for Reporting Qualitative Research (COREQ) checklist [25].

### Participants and data

Participants were partners of the research project recruited to the study via email request and gave informed consent. All were involved in the development of the Navigator and had diverse educational backgrounds including economics, nursing, social work, administration, pharmacy, and medicine. Two researchers (A-M.M-K. and T.K.) conducted the interviews on Microsoft Teams, with only the interviewers and interviewees present. There were ten interviews with eleven participants. Interviews were audio-recorded and transcribed verbatim. Transcriptions were not checked by participants. The interview schedule is presented in Appendix. Field notes were not used. The interviews ranged from 43 to 103 min in length (*M* 64.3; *SD* 17.8).

## Method

Data analysis followed the process of inductive content analysis: determining the research questions, selecting the data, constructing a coding frame, conducting segmentation, conducting trial coding, refining the coding frame, conducting the main analysis, and finally presenting and interpreting the results [26]. J.K. conducted the preliminary analysis. This involved data familiarisation through reading the texts thoroughly before coding transcripts by content in the second reading round. These were then condensed into a thematic map with two pre-determined overarching main themes on ATLAS.ti version 23.1.1.0. These were 1) reflections on individuals with SUD as the end users of digital solutions, and 2) substance use workers as the end users of digital solutions. Citations were extracted from the text and organised separately based on the coding structure. The subsequent reading and coding revealed a range of subthemes from the remaining codes (Table 1). The subthemes were decided inductively based on significance, repetition, and consistency in the data. The aim of inductive content analysis is to construct descriptive knowledge and deeper understanding [27]. Inductive approach focuses on revealing systematic patterns which enables finding consistent similarities and differences in the data [26, 28].

**Table 1:** The themes and subthemes from the content analysis of digital service development'  professionals thoughts on digital solutions and clients of substance use services

Main theme	Sub-themes
**1. Reflections on individuals with SUD as the end users of digital solutions (*n* = 86 citations)**	1.1 Support needs
	1.2 Digital divide
	1.3 The nature of problematic substance use
	1.4 Stigma
	1.5 Facelessness and anonymity
	1.6 Increased freedom of choice
**2. Substance use workers as the end users of digital solutions (*n* = 140 citations)**	2.1 Change
	2.2 Resources
	2.3 Deployment and management
	2.4 Interdisciplinary co-operation
	2.5 Digitalisation

## Results

From ten interviews with eleven professionals who were involved in the development of the Navigator, we examined their perceptions of people with problematic substance use and substance use workers as the end users of digital solutions. Results are presented as: 1) *The suitability and effectiveness of digital solutions for individuals with SUD and related challenges* and 2) *The deployment of digital solutions in substance use work.* The sub-titles represent the sub-themes introduced in Table 1. The citations are marked with numbers representing I = interview, and in the case of interview 1 with two participants (P1 or P2).

### The suitability and effectiveness of digital solutions for individuals with SUD and related challenges

#### Support needs

Participants identified substance users as a heterogenous group of people. They understood support needs as individual and considered the potential to benefit from digital solutions to be similar in addiction compared to any other client group:

*"That's what I said, whether drug users are thought of too much as drunkards [rapajuoppo] or drug addicts on that side of the market, but maybe drug users are different as well as in all other customer or patient groups. And there are certainly those who can and would benefit from digital services” (P2 in I1)*

*“...I don’t see that they [people who use substances] are any different from any other client group.” (P1 in I1)*

Although participants viewed people with SUD as a heterogenous group with individual needs, digital solutions were mainly targeted to those clients who had the resources to access and use them. For others, traditional face-to-face services were provided. This reveals two important things. Firstly, when talking about digital solutions, the most vulnerable clients are excluded very quickly. Secondly, to ensure equitable service access, the need for traditional face-to-face services persists alongside digital solutions.

#### Digital divide

According to the data, designing digital solutions for all those with SUD requires attention and a willingness from providers. Otherwise, individuals in the most vulnerable positions are at risk of exclusion from services particularly if traditional face-to-face services are being replaced with digital solutions. There seems to be the potential for gaps in the service system. Unequal access to digital tools and services, or *digital divide*, was identified as a significant challenge among the participants. There were concerns regarding the engagement with digital platforms:

*"With this experience of adult social work, I can say that a big challenge was that the clients always had their phones lost, stolen, broken, and they couldn't afford to buy a prepaid subscription. This kind of concreteness comes down to how you can utilise digitalisation if you don't have the tools and connection." (I9)*

In the data, digital divide was emphasized by participants with a background in the social welfare sector. They expressed concerns regarding the lack of adequate devices, connectivity, credentials, as well as the limited language or digital skills among the most vulnerable clients. Although more visible in the social welfare sector, it is important to understand the consequences in the healthcare sector. Despite serious concerns raised, no concrete solutions to address these challenges were suggested in the interviews.

#### The nature of problematic substance use

Another challenge within this clientele was the complexity of the life situations of the individuals with SUD. Here, the participants discussed the social dimensions with addictions:

*"I always wonder why Jeppe drinks [reference to the work of Ludvig Holberg – Jeppe on the Hill; Or, The Transformed Peasant], what is the basic reason, what is the root cause. So, I think that it is often these social questions, like what is the everyday life of a person like, what are the social contacts, or are there any at all, how lonely is he, and is there anything to do, is he in work life... And financial matters, they are really important. When it comes to substance abuse problems, I think that the health part is actually quite small after all." (I5)*

*"My own experience was at least that most of the time there was also a really strong financial aspect, that there is something like that, that needs to be taken care of in order to be able to go to rehabilitation, so that after rehabilitation you can orientate yourself again. And that's what has to be thought out, so that the client does not return to the streets." (I9)*

Participants claimed that the social welfare and healthcare system often fails to identify unhealthy substance use, resulting in delayed access to services. Consequently, by the time clients’ access care, their life situations could have become more complex. Substance use disorder was perceived as involving multiple interconnected problems rather than being a single, isolated concern. The participants stressed the importance of comprehensive treatment, emphasizing the need to address the client’s entire life situation rather than focusing solely on individual problems or symptoms. They emphasized the importance of participation, involvement, freedom of choice, and establishing a partnership, all of which helped clients to successfully achieve their treatment goals.

*“Well, it's just this, since this [the Navigator] has its own logic that clients are involved in this, at least as a client I would appreciate it, that hey, I can actually somehow influence these services, what I get and what are good for me." (I3)*

*"And the biggest change in digital services is that the client should take part in the service production, i.e. the client produces some part of the service planned for them themself." (P2 in I1)*

Some participants thought clients should contribute to the care plan, however, not all interviewees shared the same optimism regarding client-involvement. Particularly for those in the most vulnerable position, expecting them to make independent and rational choices within the care system was thought not to be feasible by some:

*"I think it's a complex issue, how do we listen to the client's experience, it's impossible to think that everyone has the resources and the desire to be involved in such development patterns, client council patterns, or expert by experience work. Like how we can get there, that we would really listen in some way systematically that human experience, that experience as a user of that service." (I4)*

There were concerns certain clients with more complex challenges would be excluded from the development process due to potential lack of interest or resources to participate. The notion clients should be responsible for articulating their situations in a manner that qualifies them for necessary services was deemed unrealistic. According to the interviews, clients in the most vulnerable situations can be hard to reach, and they may face obstacles in committing to the treatment, some of which may not be of their own making. As such they may need additional support and understanding:

*“This client group is a bit more challenging when it comes to treatment." (I10)*

*"When the crisis had been taken care of, even if the next meeting time was agreed upon, the client didn't necessarily show up, maybe they weren't ready to commit to anything at that point. I think that [the Navigator] would be more suitable for those clients who are perhaps more committed to treatment or maybe for those proceeding in opioid-maintenance treatment. Maybe it could have been used for these clients at some point, but not at that point when the client's commitment to [names of the low threshold substance use services] was occasional, so that the client could maybe pick up the medicine or visited when there was some acute matter to be taken care of.” (I9)*

#### Stigma

According to the participants, reaching out particularly through digital means, can pose significant challenges. This was perceived to be difficulties in client commitment to services or treatment, coupled with obstacles in reaching them, e.g., lack of mobile devices or a stable address. Numerous barriers and substantial challenges were described in availability and accessibility. The threshold for accessing services and treatment may be elevated due to the experienced or feared stigma and biased attitudes:

*"But it [social work] becomes precisely the question of deserving and undeserving. It's a question of, well, it is your own fault that you messed up your life by yourself. And so, in general, the fact that the client floods have been managed with certain thresholds. It has become an “agility track” to even getting into social work. Like, go over and go under and go between, and maybe you'll get it [social work]." (I4)*

*"The person may wonder, what are the consequences, if I answer [the questions of the Navigator]. If drugs are involved, then maybe this person thinks that will this lead to something like, is my boss being contacted, if the person is in working life. Probably something related to shame or something, that this person doesn’t want this information to spread.” (I3)*

Based on the interviews, stigma and biased attitudes occur in society, among professionals in social welfare and healthcare and in their structures. According to some participants, clients are expected to prove their eligibility for services and treatment through active engagement. Biased attitudes perceive problematic substance use as self-inflicted. Stigma generates feelings of shame, leaving individuals to conceal their problems rather than seek help.

#### Facelessness and anonymity

Alongside the identified challenges, the participants also recognized benefits of digital solutions for individuals with SUD. One recognized benefit was the anonymity and impersonal nature of digital services, which facilitates more honest responses without the immediate fear of judgement from a healthcare or welfare professional:

*“Now that the AUDIT [Alcohol Use Disorders Test] test is filled digitally, people seem to drink more, when speaking of alcohol, so it seems that we are more honest digitally than face-to-face with the professional. There is suddenly no social pressure [laughs], so people are more open.” (I8)*

Based on the data, facelessness and anonymity associated with digital platforms may reduce the experienced pressure during interaction. It seems digital environments, being more neutral spaces, can potentially alter established power dynamics, particularly if some of the other challenges (e.g. digital divide, or complex life circumstances) are not present. According to the participants, accessing digital services requires not only suitable devices, but also control, mental resources, and proficiency.

#### Increased freedom of choice

**Fig. 1:**
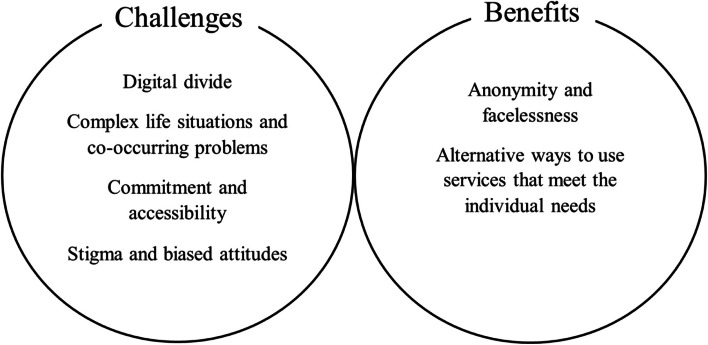
Challenges and benefits of digital solutions for people with SUD

Those capable of accessing digital services can benefit from the alternative service delivery methods they offer. Although the need for face-to-face services remains on some occasions, digital solutions were recognized as essential supplementary tools in the service provision among the participants. The identified challenges and benefits of digital solutions for addictions treatment are presented in Fig. 1.

### The deployment of digital solutions in substance use work

#### Change

In the realm of social welfare and healthcare, participants see that digitalisation entails adaptation and a need to re-learn traditional approaches to work:

*“From the perspective of professionals, digitalisation means primarily a change in work, and it is the most difficult thing in this, that digitalisation should not be considered just as some peripheral solution, but part of the work changes concretely by it. The best or the worst phenomena in it can be working from home, the breakdown of work communities... Especially in social welfare and health care work, the technology and digital skills, and maybe the management of some kind of everyday problem-solving skills are strongly related.” (I8)*

According to the participants, digitalisation cannot be understood as an add-on solution; it is a change in work processes requiring re-learning and potentially adding to the cognitive load at work. It may impact professional work communities and/or foster an increase in remote work.

#### Resources

The participants recognized that professionals may face obstacles in accessing and utilising digital solutions. Accessibility was deemed important for workers in addition to clients. Many digital solutions necessitate professionals to learn and maybe undergo training before implementation. Based on the interviews, due to highly regulated information security and confidentiality regulations, professionals should be granted time to familiarise themselves with new digital solutions before implementing them into practice. Scarce resources are characteristic of public sector care system. According to the interviews, the lack of resources seemed to be the most critical challenge when it comes to the deployment of digital solutions in public care system:

*“The most critical resource is by no means always the money, but at the moment we live in such a time that the more critical resource is the employees.” (I8)*

*“It is quite difficult to try anything new or to pilot anything new if for example at some healthcare station five full-time nurses are lacking and also on the social welfare, some units giving counselling and guidance had to be closed due to long work absences.” (I9)*

According to the participants, the lack of a competent workforce was identified as the most crucial issue within the field presently. Participants expressed difficulty implementing new solutions amid the persistent staff shortage, leading to feelings of increased burden and constant time pressure. To successfully deploy a new digital solution, professionals needed a clear understanding of how this solution could potentially benefit both them and their clients:

*“Probably some concreteness is called for. -- But I think that the people in the field, if they could see, that something is beneficial and really for example somehow reduces clients’ service use in that unit, if that is what is being wanted, or if reducing the number of phone calls is being wanted or increasing good experiences among clients somehow. All that, that makes the results visible, that why is this being used.” (I9)*

#### Deployment and management

Based on the interviews, if the benefits of digital solutions remain unclear for the professionals, their resistance to deployment tends to increase and instead it is perceived as an additional burden rather than a tool that simplifies their tasks. However, some participants highlighted professionals’ tendency to prioritise their own perspective; some professionals may be more inclined to consider personal benefits rather than client benefits. The participants called for a shift toward a more client-oriented thinking among field workers regarding digital solutions:

*“I feel that the professionals quite much think about those things just from the perspective of a professional, like how can I benefit from this and what can I get from this. It is not just that, that how can I help the client the best way, even if they would want to, but has maybe become somehow numb or is always in such a hurry, that doesn’t have time, that now I only do what’s the easiest for myself.” (I3)*

Some participants perceived implementation challenges as a management issue in the deploying organisation sometimes lacking strength and consistency. While it may have been easy to persuade management to acquire the digital solution, the responsibility for executing the deployment fell on the organisation’s staff. Some of the participants emphasized that the deployment cannot rely on external forces or be executed from outside the organisation. They brought up that in social welfare and healthcare, the dialogue between the management and operational staff appeared to be weak or even non-existent. Internal technical difficulties may also have hampered the deployment process.

#### Interdisciplinary co-operation

According to the interviews, field professionals often lack technological proficiency and require technological expertise. In this domain, collaborative development involving various stakeholders was deemed crucial:

*“I see that multi-professionalism is what is needed today, that social welfare and healthcare sector won’t make it with social welfare and healthcare professionals among themselves. On the other hand, technology won’t make it with technology experts among themselves either. So, in my opinion, it is great that today there is an understanding that we develop the whole service system, the whole social welfare and health care sector, multi-professionally. (I2)*

The participants emphasized that field workers possess the most up-to-date insights into clients’ needs as they encounter them daily. They can effectively evaluate whether digital solutions adequately cater to these needs. According to the participants, involving professionals in the development and post-deployment phases may alleviate resistance to change among the personnel.

In Finland, the social welfare and healthcare reform in 2023 aimed to streamline services by transferring responsibility from over 300 municipalities to 21 wellbeing services counties. This transfer was intended to enhance collaboration between social welfare and healthcare sectors. Nevertheless, the participants recognised remarkable disparities in the structures of services and processes between social welfare and healthcare:

*“In my opinion, a very big problem in social work which is visible in for example government’s research funding, is that how much the medicine receives [funding], has received in 30 years how many millions a year, and social work has received [funding] in the last three years four millions, or what it was. So that in a sense, there is an idea, that in social work, research, development, and all this happens without anything, or along everything else.” (I4)*

Based on the interviews, differences in the structures and processes between social welfare and healthcare sectors may hamper collaborative advancement. The participants described that digital solutions tailored for healthcare may not seamlessly apply to social welfare, and vice versa. The participants with a background in social work highlighted that social work often remained in the shadow of healthcare when it comes to development, decision-making, or funding. One participant described collaborative groups with numerous healthcare representatives but only one from social services, still being considered as multi-professional co-operation. In the light of the data, when designing digital solutions for both domains, equal involvement and sufficient resources for both areas of expertise are important.

The participants noted that professionals leading digital solution co-development are typically those particularly interested in digitalisation. They stated that field workers who may resist change, may be more difficult to engage in the co-development process, and might skew developers’ perception of the field’s receptivity to digital solutions.

*“Surely, those who participate in our co-development too, are people with a positive attitude towards digitalisation and who can see how these digital tools as results of digital development can benefit in practice. We may encounter more seldom those who have decided that “I won’t use this, no matter what, I won’t”. [Laughs] I guess we only see the most digitally oriented peak.” (P2 in I1)*

#### Digitalisation

Participants noted that digitalisation has the potential to drive the standardisation of service delivery, a concept supported by many. They deemed digitalisation could streamline client work, enhancing efficiency by minimising human errors, and promoting equitable treatment. In an era marked by chronic labor shortage, improving efficiency was deemed crucial. However, concerns were raised the focus of digitalisation leans more towards standardising client work rather than enabling client-centered approach. Participants saw there is a risk that digitalisation prioritizes system-orientation over client-orientation, conflicting with contemporary client-centered practices. Although digital solutions aim to enhance work efficacy, they seemed to escalate the perceived workload among professionals:

*“And then I’ll raise this issue that has bothered me for a long time, that when we create those digital services and channels, like chat and [a digital health care application], and what else is coming, when there is a need to build communication platforms between the wellbeing services counties and municipalities. The point, that how many digital channels co-exist, that should be tried to manage simultaneously in the public sector. And then if there is a staff shortage, so which channel is the number one priority to answer to, is it the phone, or is it the personal face-to-face service, that there must be someone answering? Do I answer the chat, if there is really a hell of a burning rush. What about those notifications coming via [a digital health care application], what is the delay of the response. Then if someone comes to the office, and then there are the emails, and what are all these other possible channels that may exist. The fact that to how many channels do I have to turn myself into when responding and reacting, so that the client would feel that they can contact us and get into a dialogue?” (I9)*

All new digital solutions require learning or re-learning of processes, which, according to the interviews, appear to face resistance in an overburdened field. The participants described how the multi-channel nature of services can also result in unnecessary contacts to the service system and create disruption demand. In addition, digital solutions are often developed alongside existing processes rather than replacing them. According to the participants, this could lead to a situation where traditional processes remain while several additional processes are introduced, consequently increasing the experienced workload. Although a single practice could reduce time consumption and enhance work efficiency, the simultaneous operation of multiple processes escalates the overall workload.

## Discussion

In this inductive content analysis of interviews with professionals involved in the development of the Navigator, we examined their perceptions of individuals with SUD and those who care for them as the end users of digital solutions. The findings highlighted both benefits (anonymous and faceless service use, alternative ways of using services that can better meet the individual needs, decreased time consumption) and challenges (digital divide, complex life situations, accessibility, commitment, stigma and biased attitudes, increased workload, service standardization) associated with digital solutions for vulnerable and stigmatised groups.

Individuals dealing with SUD were acknowledged as a heterogenous group with unique situations and needs (see also [29]). Experiences with stigma can lead to the clients’ inability to engage [12]. This is not unique to treatment for those with SUD; many aspects of service provision for this client group are compromised by stigma e.g., naloxone provision [30, 31]. Challenges like the digital divide, experienced or anticipated stigma and shame, complex life circumstances, and difficulties in committing to treatment were identified as barriers to digital service utilisation. In turn, digital solutions were perceived as avenues that could offer alternative and more tailored services to address client’s individual needs. Client-oriented approach was called for, advocating for increased client involvement in service development and production. This sits uncomfortably alongside the stigma which disempowers clients [8, 32, 33].

For substance use workers, digitalisation implied a comprehensive change, requiring re-learning of familiar work processes. This increased cognitive burden under already stressful conditions. Like advances in AI for health and social care it will be important to monitor the tool for accuracy and quality care [34]. Participants advocated for a multi-professional approach, highlighting existing inequalities in funding and representation between social services and healthcare. Contrary to its intended purpose of reducing workload and enhancing efficacy, the findings suggested digitalisation might, in fact amplify workload by introducing additional co-existing working channels. Managing various simultaneous working channels such as phones, chat, client information system, email, and other possible digital channels on top of face-to-face encounters seem to challenge today’s client work and requires some innovation in workplace design and practice [35]. To achieve the benefits for their clients, the professionals must be given time to learn and internalise new digital solutions prior to use.

Interestingly, while developers called for more client-centered thinking among the professionals, they also supported service standardisation. According to Engström and colleagues [25] service standardisation increases the risk for clients in the most vulnerable situations to fall outside the service system. Service standardisation increases system-oriented thinking and managerialism in client work which prevents client-oriented practice [36]. System-oriented thinking leads to service provider orientation in treatment which has been criticized [24]. Involving clients in decisions about their care is the best practice [18, 37, 38]. Concerns about how to implement new digital solutions may relate to concerns about how much work is involved, rather than disinterest in prioritising client orientations.

Ultimately, the digital revolution is an opportunity to change services to reduce stigma and biased attitudes and improve healthcare outcomes [39]; people should no longer encounter stigma in the care system and this study aligns with previous works to illustrate that biased attitudes still persist (e.g., [8, 10–12]. People with SUD were seen as hard to reach and difficult to commit to the treatment. This observation is in line with previous research while treatment seeking rates have typically been low and treatment dropout rates high among people with substance use problems [22]. However, pointing out these issues to the client instead of the stigmatising and discriminatory structures of the care system can prevent us from seeing the root causes and taking responsibility and ultimately improving care through effective partnership [40].

For some, digital solutions were seen to lower the threshold to access the services due to reduced fear of stigmatisation which is in line with previous observations [13, 15]. Availability and anonymity have been observed to be important factors for choosing to use online-based services [41]. However, anonymity or lower threshold to services will not help in the long run if the system-level structures (e.g., language and attitudes) maintain stigma. It is essential for the public care system to actively address stigma and prevent reproducing it, fostering a safe environment to access services and seek help.

User-involvement and a firm evidence base are vital when designing and implementing digital solutions for stigmatised groups [15, 18]. There is a risk that digital development leads to digital inequality and deepens the digital divide [14]. Implementing new digital interventions for stigmatised groups without sufficient evidence base can, at worst, jeopardize clients’ position in the service system. The challenges identified in the interviews of those involved in the development of the Navigator indicate that there may have been too little evidence before implementing the tool for this clientele.

This study has a couple limitations to be mentioned. One limitation is that the data was collected from experts who were all involved with the same digital solution, the Navigator, rather than multiple or different digital solutions. However, the interviews revealed valuable information on how digital solutions may be adopted in other fields beyond SUD and other stigmatised groups. Also, the study participants represented various educational backgrounds, enriching the data. The results of this study have several implications in both public care system practices and the practices of companies developing digital services for public care purposes. Findings can support developing, planning, implementing, or deploying digital solutions for a range of different target populations and especially for stigmatised groups in health care and welfare. For future research this study calls for including end users in the discussion of digitalisation, efforts to reduce stigma, and overcoming the digital divide [42].

## Conclusions

Digitalisation will continue to shape health and welfare services internationally to reduce costs and maximise resource use. People with problematic substance use and professionals who help them were seen to face some special challenges to implement digital solutions. Digital divide, stigma, and complex situations along with chronic staff shortages, cognitive burden, and service standardisation were factors that limited the deployment of digital solutions among this clientele. Digital solutions must have a strong evidence base before wider implications, especially when they are implied for stigmatised and vulnerable groups. The question of how to secure the participation and involvement of those in the most vulnerable position in digitalisation remained unanswered. In the future answers are needed when digital processes become more common and non-digital processes are continuously being run down.

## Data Availability

No datasets were generated or analysed during the current study.

## Appendix

**Table:** Semi-structured interview questions to understand digital service development professionals views on client segmentation and individuals with substance use disorders

Theme Interview Framework		
	**Main themes**	**Sub-themes**
**1**	Definitions of the effectiveness of social welfare and health care services and discussions regarding them in relation to social services, including substance use services	a) Effectiveness in general and in substance use services in particular
		b) Consideration of different aspects of effectiveness (costs, work efficiency, client impact)
**2**	Decision-making and measurement related to effectiveness in social services, including substance use services	a) Decision-making on effectiveness measures
		b) Measuring and verifying the results of effectiveness measures
**3**	Operative methods, models, and tools in promoting the effectiveness of substance use and social services	a) Models/tools implemented to promote effectiveness
		b) The benefits and challenges of these models/tools
**4**	Prerequisites for client segmentation according to service needs in the municipality	a) Client segmentation from the perspective of the municipality’s service processes
		b) Client segmentation from the perspective of the operating area and the characteristics of the services provided
**5**	Experiences of using the digital client segmentation tool with clients who use substances	a) Deployment and related decision-making
		b) Practices, technical conditions, and information security
		c) Benefits and challenges of using the tool
		d) Communication structures and experience of shared expertise
**6**	Clients’ view of the effectiveness of the service and using the digital client segmentation tool	a) Effectiveness of the services provided for problematic substance use
		b) The digital client segmentation tool user experience and added value
		c) Experience in participation, communication, and shared expertise

## Abbreviations

SUD: Substance use disorder
AUDIT: The alcohol use disorders identification test
